# Effect of the 3D Artificial Nichoid on the Morphology and Mechanobiological Response of Mesenchymal Stem Cells Cultured In Vitro

**DOI:** 10.3390/cells9081873

**Published:** 2020-08-11

**Authors:** Andrea Remuzzi, Barbara Bonandrini, Matteo Tironi, Lorena Longaretti, Marina Figliuzzi, Sara Conti, Tommaso Zandrini, Roberto Osellame, Giulio Cerullo, Manuela Teresa Raimondi

**Affiliations:** 1 Department of Management, Information and Production Engineering, University of Bergamo Viale Marconi 5, 24044 Dalmine, Italy; 2 Department of Chemistry, Materials and Chemical Engineering “Giulio Natta”, Politecnico di Milano, Piazza Leonardo da Vinci, 32, 20133 Milano, Italy; bonandrinib@gmail.com (B.B.); tommaso.zandrini@polimi.it (T.Z.); manuela.raimondi@polimi.it (M.T.R.); 3 Department of Biomedical Engineering, Istituto di Ricerche Farmacologiche Mario Negri—IRCCS, Via Stezzano 87, 24126 Bergamo, Italy; matteo.tironi@marionegri.it (M.T.); marina.figliuzzi@marionegri.it (M.F.); 4 Department of Molecular Medicine, Istituto di Ricerche Farmacologiche Mario Negri—IRCCS, Via Stezzano 87, 24126 Bergamo, Italy; lorena.longaretti@marionegri.it (L.L.); sara.conti@marionegri.it (S.C.); 5 Istituto di Fotonica e Nanotecnologie (IFN)—CNR and Department of Physics, Politecnico di Milano, Piazza Leonardo da Vinci, 32, 20133 Milano, Italy; roberto.osellame@polimi.it (R.O.); giulio.cerullo@polimi.it (G.C.)

**Keywords:** mesenchymal stem cells, 3D culture, artificial niche, mechano-transduction, nuclear pore size

## Abstract

Stem cell fate and behavior are affected by the bidirectional communication of cells and their local microenvironment (the stem cell niche), which includes biochemical cues, as well as physical and mechanical factors. Stem cells are normally cultured in conventional two-dimensional monolayer, with a mechanical environment very different from the physiological one. Here, we compare culture of rat mesenchymal stem cells on flat culture supports and in the “Nichoid”, an innovative three-dimensional substrate micro-engineered to recapitulate the architecture of the physiological niche *in vitro*. Two versions of the culture substrates Nichoid (single-layered or “2D Nichoid” and multi-layered or “3D Nichoid”) were fabricated via two-photon laser polymerization in a biocompatible hybrid organic-inorganic photoresist (SZ2080). Mesenchymal stem cells, isolated from rat bone marrow, were seeded on flat substrates and on 2D and 3D Nichoid substrates and maintained in culture up to 2 weeks. During cell culture, we evaluated cell morphology, proliferation, cell motility and the expression of a panel of 89 mesenchymal stem cells’ specific genes, as well as intracellular structures organization. Our results show that mesenchymal stem cells adhered and grew in the 3D Nichoid with a comparable proliferation rate as compared to flat substrates. After seeding on flat substrates, cells displayed large and spread nucleus and cytoplasm, while cells cultured in the 3D Nichoid were spatially organized in three dimensions, with smaller and spherical nuclei. Gene expression analysis revealed the upregulation of genes related to stemness and to mesenchymal stem cells’ features in Nichoid-cultured cells, as compared to flat substrates. The observed changes in cytoskeletal organization of cells cultured on 3D Nichoids were also responsible for a different localization of the mechanotransducer transcription factor YAP, with an increase of the cytoplasmic retention in cells cultured in the 3D Nichoid. This difference could be explained by alterations in the import of transcription factors inside the nucleus due to the observed decrease of mean nuclear pore diameter, by transmission electron microscopy. Our data show that 3D distribution of cell volume has a profound effect on mesenchymal stem cells structure and on their mechanobiological response, and highlight the potential use of the 3D Nichoid substrate to strengthen the potential effects of MSC in vitro and in vivo.

## 1. Introduction

Cell therapies represent one of the most promising approaches in medicine. The potential use of stem cells has increased expectations for repairing organ damage and replacing diseased tissues. The most feasible application of stem cell therapy involves the use of adult stem cells, such as mesenchymal stem cells (MSCs), since these cells are not limited by ethical issues and can be easily obtained from adult tissues such as adipose tissue and bone marrow (BM). Once implanted in a recipient, MSCs can secrete potent combinations of trophic and immunomodulatory factors that are able to modulate several responses in surrounding cells and in circulating cells [1,2,3,4,5,6]. In addition, the secretion of several bioactive factors, which play an important role in the regulation of physiological processes, makes the use of MSC secretome an attractive cell-free alternative for regenerative medicine approaches [7,8,9]. In the BM microenvironment, MSCs are in close association with hematopoietic stem/progenitor cells and are thought to have an important supportive role due to the secretion of hematopoietic cytokines [10,11]. The function of MSCs in the natural niche, as well as of those residing in another tissue after cell transplantation, crucially depends on biochemical as well as biomechanical signals. The microenvironment that surrounds stem cells in the BM, called the stem cell niche, provides a variety of physical signals. This complex interplay between mechanical constraints and the resulting changes in cell functions is not completely understood so far. A large body of evidence suggests that mechanical properties of MSC substrates determine cell proliferation and differentiation, as well as important changes in gene and protein expression [12,13,14,15,16,17,18,19]. It is not clear at the moment which is the selective effect of three-dimensional (3D) disposition in the space on MSC biology. It has been suggested that MSC in the bone marrow niche form a micrometer-sized 3D network in contact with the hematopoietic/progenitor stem cells [20]. Even when MSCs are cultured in vitro, physical and mechanical factors, including substrate stiffness, surface nano-topography, and extracellular forces all play a crucial role in triggering cell functions [21,22,23].

Conventional cell culture used to investigate stem cell behavior is often restricted to two-dimensional (2D) cell monolayers. However, 2D cultures strongly alter cell biological functions, as they do not reproduce the complex 3D environment of the stem cell niche. In addition, 2D substrates confine cells to a thin planar layer, in which mechanotransduction processes are strongly different from those present in a 3D culture environment. Two-photon polymerization (2PP) is a nanofabrication technique that allows precise control of the scaffold geometry in 3D, with a feature size comparable to or even smaller than the characteristic cellular size. This technique is based on the simultaneous absorption of two or more infrared photons of a femtosecond pulsed laser tightly focused inside a UV photosensitive resist, that leads to polymerization only in the laser focal volume. The nonlinearity of multiphoton absorption causes a high confinement of the polymerized region along the laser beam axis, and the threshold mechanism that regulates photo polymerization results in a spatial resolution of ≈100 nm, which is below the light diffraction limit, enabling the ultraprecise fabrication of arbitrary 3D geometries. However, the serial nature of the 2PP process limits its use for large-scale fabrication, so it is usually employed for structures covering a few hundreds of microns [24]. In our work, we started from a base structure called “3D Nichoid”, of dimension of around 100 microns, and we worked on the optimization of the fabrication process to be able to pattern the widest possible culture area with it. In the last five years, we have been able to pass from substrates with just a few Nichoids, to the substrates of around 1 cm^2 in area, covered by eight thousand 3D Nichoids [25], as those used here.

The 3D Nichoid culture substrate confines adhering cells to functional microenvironments shaped as microscopic grids, in which MSCs migrate and home spontaneously [26], proliferate faster [27] and maintain a significantly greater stemness compared to monolayer-cultured cells [28]. The general aim of the present work then was to investigate to which extent the culture of MSCs on the up-scaled version of the Nichoid micro-engineered substrate affects cell structure and function, as compared to 2D cell culture, in terms of expression of transcriptional factors, gene regulation and cell stemness. We investigated specifically whether mechanotransduction triggered by the 3D micro-structure may be responsible for changes in cell biology, independently from biochemical conditions. We hypothesize that the architecture of the 3D Nichoid microstructure induces a cell adhesion configuration, capable of modifying MSCs nuclear envelope and the nuclear pore structures. Such conformational alterations could be responsible for changes in nuclear import of key molecules, such as transcription factors, that determine the differentiation fate of MSCs.

As a matter of fact, it has been reported that forces transmitted from the cell surface to the nucleus by the cytoskeleton, result in chromatin stretching and changes in gene transcription [29]. Through these processes, mechanical signals can direct MSCs’ fate and behavior even in the absence of specific changes in chemical signals [21,30]. One of the most important effects of this mechanotransduction mechanism is the activation of YAP and TAZ, which are involved as downstream elements in cells perception of the physical microenvironment. While this phenomenon has been demonstrated for substrates having different mechanical properties, it has not been studied so far for 3D substrates used to mimic the stem cell niche in cultured MSCs. Here we investigate the structural and functional differences of MSCs cultured on 2D surfaces and in the 3D Nichoid micro-structured grid. We specifically estimate whether the different spatial conditions may induce changes in MSC biology that affect cell stemness, gene and protein expression.

## 2. Materials and Methods

### 2.1. Fabrication of Nichoid Culture Substrates

**Figure 1:**
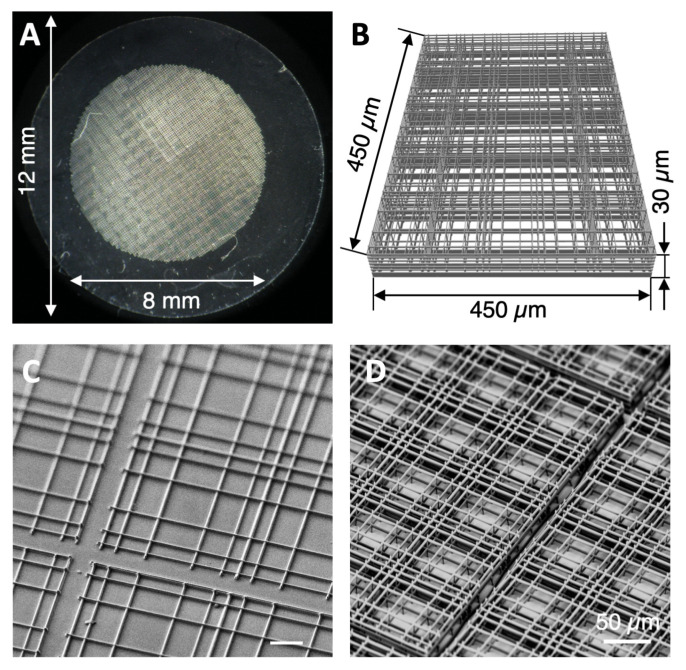
The Nichoid culture substrate fabricated by two-photon laser polymerization (2PP). (**A**) Photograph of the Nichoid synthetic niche culture system. The structure is composed by a matrix of 218 elementary blocks. (**B**) CAD of one elementary block of the microfabricated 3D Nichoid structure. (**C**) Scanning electron microscopy (SEM) image showing the 2D Nichoid (single layer). (**D**) Adjacent blocks of the 3D Nichoid are separated by small spacing of 30 µm between each other.

The micro-structures used for cell culture in this study, later referred as “Nichoid” substrates (Figure 1A), were fabricated by 2PP as previously described in detail [25]. The biocompatible photopolymer SZ2080 was used with 1% of Irg photo-initiator (Irgacure 369, 2-Benzyl-2-dimethylamino-1-(4-morpholinophenyl)-butanone-1). The laser used for 2PP was a cavity-dumped Yb:KYW system producing pulses with 300-fs duration and 1-MHz repetition rate (1030 nm wavelength) focused with a 1.4 numerical aperture (NA) oil immersion objective (Carl Zeiss, Oberkochen, Germany). Computer-controlled, three-axis motion stages (ANT130, Aerotech, Pittsburgh, PA, USA) were used to control sample motion around the laser focal spot. A total of 218 elementary blocks of niches were laser written directly onto circular glass cover slips (150 µm thickness and 12 mm diameter, BioOptika, Milan, Italy). As shown in Figure 1, two types of elementary blocks were fabricated. One with a single-layered geometry (later referred to as 2D Nichoid) consisting of single interconnected rods (450 µm wide and 450 µm long) forming 25 repetitive units (90 µm × 90 µm), as shown in Figure 1C. The second type of Nichoid, the 3D one, consisting of similar 25 repetitive units (30 µm high, 450 µm wide and 450 µm long) composed of 25 units (30 µm high and 90 µm × 90 µm in transverse dimensions) consisting of three piled up layers, each with the same architecture of the 2D Nichoid, made of interconnected rods, with a graded transverse spacing between 10 and 30 µm, and a uniform vertical spacing of 15 µm (see Figure 1B,D). The spacing between adjacent blocks was set to 30 µm, as shown in Figure 1D.

### 2.2. Isolation of Rat MSCs

All animal studies were approved by the Institutional Animal Care and Use Committees of the Mario Negri Institute. Animal care and treatment were conducted in accordance with the institutional guidelines, in compliance with national (D.L.n.26, 4 March 2014), and international laws and policies (directive 2010/63/EU on the protection of animals used for scientific purposes). All rats were maintained in a pathogen-free facility with a 12-h light/dark cycle and free access to standard diet and water. Six-week-old male Sprague Dawley rats (Charles River Laboratories Italia, Milano, Italy) were euthanized by CO_2_ overdose and bone marrow aspirates were collected by flushing the femurs and tibias with cold medium. After being filtered through a 100 µm sterile filter (BD Biosciences, Milan, Italy), bone marrow cells were plated in α-MEM (Invitrogen, Waltham, MA, USA) supplemented with 20% FBS (Invitrogen), 100 U penicillin and 100 µg/mL streptomycin (Invitrogen) and allowed to adhere at 37 °C in a humidified atmosphere containing 5% CO_2_. After 72 h, non-adherent cells were removed by changing the medium, and the plate was washed twice with PBS. The medium was replaced every 3–4 days thereafter. When cultures were near confluence, cells were detached with 0.05% trypsin-EDTA solution and cryopreserved in liquid nitrogen. MSCs were characterized for their capability to differentiate toward osteoblasts and adipocytes after exposure to osteogenic and adipogenic media, as shown in Figure S1, Supplemental Information (A and B). In addition, MSC phenotype was confirmed by surface marker expression analysis. The results are reported in Figure S2, Supplementary material. All experiments were performed with cells at passage 2–4.

### 2.3. Substrate Preparation and MSCs Culture

Control glass coverslips and Nichoid substrates (both 2D and 3D) were washed thoroughly in deionized water and treated with 70% ethanol for 2 h, dried and UV-sterilized. Each sample was positioned inside a well of an Ultra-Low Attachment 24 multi-well plate (Costar 3473, Corning, NY, USA). MSCs were seeded at concentration of 1–2 × 10^4^ cells/cm^2^ on the coverslips and on the 2D and 3D Nichoid substrates, and maintained in culture for up to 2 weeks. During culture, cell adherence and growth were monitored and assessed using a standard inverted microscope (Axiovert 40 C, Carl Zeiss Inc., Göttingen, Germany) equipped with a digital camera (PowerShot G5, Canon Inc., Tokyo, Japan).

### 2.4. Scanning Electron Microscopy

For scanning electron microscopy (SEM) observation, cells cultured on the different substrates were fixed in 0.5% glutaraldehyde for 1 h at 4 °C, washed in cacodylate buffer, and then post-fixed with 1% OsO4 for an additional hour. Fixed specimens were dehydrated through a series of passages in increasing ethanol baths and dried in pure hexamethyldisilazane (HMDS, Fluka Chemie AG, Buchs, Switzerland). Finally, samples were mounted on stubs, coated with gold in a sputter coater (Agar Scientific Ltd., Stansted, England) and then examined on a Cross-Beam 1540EsB electron microscope (Carl Zeiss Microscopy, Oberkochen, Germany).

### 2.5. Cell Proliferation

Cell proliferation was evaluated by assessing the number of cells on fixed samples at day 1, 7 and 14 of culture, after nuclear counterstaining with DAPI. For each sample, the number of cells/field was manually counted in at least 20 random fields, and the total number of cells/mm^2^ was estimated. The number of cell doublings during the two time intervals between day 1 and 7 and between day 7 and 14, was also calculated.

### 2.6. Analysis of Cellular Motility

MSCs were plated on control flat substrates or in the 2D and 3D Nichoid at a density of 1 × 10^4^ cells/sample. After 24 h, cell nuclei were stained by Hoechst nucleic acid stain (Invitrogen) for 10 min at 37 °C. After washing, movements of the adherent cells were recorded by placing seeded substrates in a Zeiss Axio observer Z1 microscope chamber (Carl Zeiss Microscopy, Oberkochen, Germany) at 37 °C with 5% CO_2_. Phase contrast and fluorescence images were taken every 5 min for 7 h with an AxioCam MRm R31 camera (Carl Zeiss Microscopy, Oberkochen, Germany). The videos were assembled with AxioVision software (release 4.8; Carl Zeiss Microscopy) and analyzed by the AxioVision Tracking module.

### 2.7. PCR Array

At the end of expansion on control (glass surface) or 3D Nichoid substrate, we investigated the expression of several genes related to MSCs biological function with the Rat Mesenchymal Stem Cell RT2 profiler PCR array (PARN-082ZC-6 SABioscience, Qiagen, Frederick, MD, USA). We investigated 89 genes encoding for MSC stemness, and MSC differentiation markers involved in osteogenesis, adipogenesis, chondrogenesis, myogenesis and tenogenesis. The complete list is reported in the Gene table, Supplementary material. For this purpose, total RNA was extracted using the RNeasy micro kit, according to the manufacturer’s instructions (Qiagen, Frederick, MD, USA) and quantified spectrophotometrically (NanoDrop ND-1000, Thermo Scientific, Wilmington, DE, USA). First-strand cDNA (1 µg) was synthesized with the RT2 First Strand Kit. Next, the cDNA was mixed with the appropriate RT2 SYBR green master mix and the resulting mixture dispensed into the wells of the RT2 profiler PCR array. qPCR was then performed on the real time cycler Viia7 (Applied Biosystems, Monza, Italy). Data analysis was performed using online SABioscience software. We then identified genes differentially expressed in the Nichoid as those affected by a twofold change (2 or 0.5) compared with the flat control.

### 2.8. Real-Time PCR

For three of the genes identified by the RT2 Profiler PCR Array, more consistently affected by the 3D Nichoid culture, mRNA expression was further evaluated with real-time PCR. Briefly, total RNA was extracted after culture on flat or in the 3D Nichoid using the RNeasy micro kit, according to the manufacturer’s instructions. During RNA extraction, contaminating genomic DNA was removed by Rnase-free Dnase treatment for 15 min. Purified RNA (2 µg) was reverse transcribed using High Capacity cDNA RT kit (Applied Biosystems). No enzyme was added for reverse transcriptase-negative controls. To amplify cDNA, TaqMan Universal PCR Master Mix (Applied Biosystems) was used according to the manufacturer’s instructions and TaqMan assays of rat LIF1 (Leukemia inhibitory factor, Rn00573491_g1), rat IL6 (Interleukin 6, Rn01410330_m1) and rat CSF3 (Colony Stimulating Factor 3, Rn00567344_m1) probes together with an endogenous control (rat HPRT1, hypoxanthine phospho-ribosyltransferase 1, Rn01527840_m1) probe. PCR assay was performed on a 7300 Time PCR System (Applied Biosystems). After an initial hold for 2 min at 50 °C and for 10 min at 95 °C, samples were cycled 40 times at 95 °C for 15 s and 60 °C for 60 s to reach the plateau. We used the ⊗⊗Ct technique (where Ct is threshold cycle) to calculate cDNA content in each sample, using cDNA expression in MSCs at day 1 as a calibrator.

### 2.9. Immunofluorescence Analysis and Quantification of YAP and pFAK

For immunofluorescence (IF) analysis, cells were fixed in 2% paraformaldehyde (PFA) in 4% sucrose. After several washes, fixed samples were permeabilized with Triton X-100 (Sigma Aldrich, Milan, Italy) and blocked with 3% bovine serum albumin. MSCs were then labeled with 2 µg/mL rabbit polyclonal anti-YAP1 antibody (Abcam, Cambridge, UK) or 10 µg/mL of anti-Rabbit phospho-focal adhesion kinase (pFAK) antibody (Abcam, Cambridge, UK) overnight at 4 °C. For YAP analysis goat anti-Rabbit IgG (H + L) secondary antibody Alexa Fluor^®^ 647 conjugate (Thermo Fisher Scientific, Milan, Italy) was used at a concentration of 2 µg/mL in phosphate buffered saline for 1 h at room temperature. Finally, cells were treated with FITC-labeled phalloidin for 45 min at room temperature and counterstained with DAPI (1 mg/mL) for nuclear staining for 15 min at room temperature. Digital images were acquired using a Nikon Eclipse Ti A1 inverted laser scanning confocal microscope (Nikon, Tokyo, Japan). Quantification of cytoplasmic YAP percentage was performed on digitized images using NIH Image J software. For each image, Regions of Interest (ROI) were selected on cell nuclei by using the DAPI signal. On the YAP signal image, the total YAP positive area was measured and then the selected ROI were applied in order to evaluate the nuclear and cytoplasmic percentages of YAP signal. For phospho-FAK analysis FITC-conjugated Anti-Rabbit IgG (H + L) secondary antibody (Jackson Immunoresearch Laboratories, Baltimore, MD, USA) was used at a concentration of 60 µg/mL in phosphate buffered saline for 1 h at room temperature. Cells were then treated with CY3-labeled phalloidin for 45 min at room temperature and counterstained with DAPI (1 mg/mL) for 15 min at room temperature.

For Western blot analysis, MSC cells cultured for 7 days on glass or the 3D Nichoid were homogenized in lysis buffer (50 mM Tris-HCl, pH 7.4), 1% Triton x-100, 0.2% Sodium deoxycholate, 0.2% SDS) containing protease inhibitors (cOmplete, Roche, Basel, Switzerland) and phosphatase inhibitors (PhosSTOP, Sigma-Aldrich) and centrifuged at 13,000 rpm at 4 °C for 10 min. Equal amounts of proteins were separated on 6–10% SDS-polyacrylamide gel electrophoresis under reducing conditions and transferred to nitrocellulose membranes (GE Healthcare, Buckinghamshire, UK). After blocking with 5% BSA (Sigma-Aldrich) in tris-buffered saline (TBS) supplemented with 0.1% Tween-20 (Sigma-Aldrich), membranes were incubated with antibodies directed against YAP1 (Abcam, Cambridge, UK), phosphorylated YAP (p-YAP, Abcam, Cambridge, UK) and tubulin (Sigma-Aldrich), followed by the appropriate secondary antibody. The signals were detected using Odyssey FC Imaging System (LiCor, Lincoln, NE, USA). Bands were quantified by densitometry using Image Studio Lite 5.0 software (LiCor).

### 2.10. Transmission Electron Microscopy

After being cultured on different substrates, MSCs were fixed in 0.5% glutaraldehyde for 1 h at 4 °C, washed in cacodylate buffer, and then post-fixed with 1% OsO^4^ for an additional hour. Fixed specimens were dehydrated through ascending grades of alcohol and embedded in Epon resin. Semithin (1 µm) sections were cut on an ultramicrotome (LKB Instruments, Bromma, Sweden), collected on a glass slide, and stained with toluidine blue. Ultrathin (100 nm) sections were cut on an ultramicrotome, collected on copper grids, and stained with uranyl acetate replacement stain (UAR, Società Italiana Chimici, Rome, Italy) and lead citrate. For the 3D Nichoid samples, cells were fixed in the scaffolds, the scaffold blocks were separated from their glass supports and semi thin sections were obtained without removing cells from the scaffolds. Ultrastructural evaluations were performed by a Morgagni 268D transmission electron microscopy (TEM) (FEI Company, Eindhoven, The Netherlands). Images of nuclear pores from the two experimental groups were taken at TEM. The estimation of pores’ diameter was performed on digitized images using NIH Image J software. Quantification was performed on several pores ranging from 80 to 100 pores imaged from samples of each group.

### 2.11. Statistical Analysis

Data were presented as mean values ± standard deviation. Differences between the groups were analyzed by Student’s *t*-test or one-way ANOVA test with Bonferroni’s correction, as recommended, and the level of significance was assumed as *p* < 0.05.

## 3. Results

### 3.1. Nichoid Structure Fabrication

The innovative 2D and 3D cell culture substrates Nichoid, fabricated by 2PP using an organic-inorganic photoresist (SZ2080), are represented in Figure 1. The photo-polymerized Nichoid micro-lattice has a Young’s modulus of 0.138 ± 0.008 GPa [24] and a porosity of 91% in terms of ratio between void volume and total volume of the structure. The result is that 88% of the available cell culture surface was covered by the 3D Nichoid [25]. The innovative 3D Nichoid substrate was used as a culture system to study MSCs adhesion and growth, morphology, proliferation, motility and gene expression in comparison to a single-layered 2D Nichoid and to a flat glass, respectively.

### 3.2. Cell Morphology

**Figure 2:**
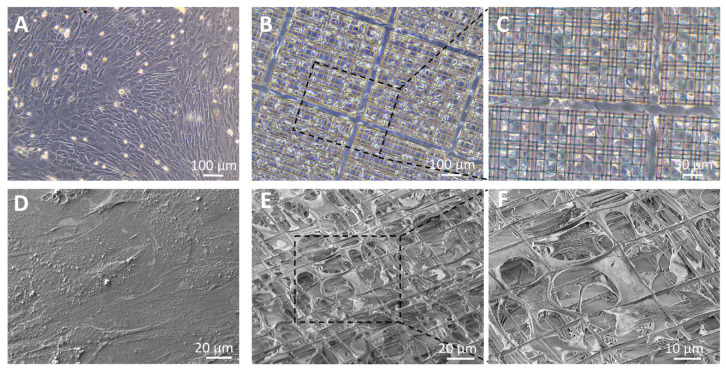
Morphological analysis of MSCs after 2 weeks’ expansion. Phase contrast images of MSCs cultured on flat control monolayer (**A**) or in the 3D Nichoid (**B**,**C**) and scanning electron microscopy (SEM) analysis of MSCs cultured on flat control monolayer (D) or in the 3D Nichoid (**E**–**F**).

We first examined MSCs attachment on different cell culture supports. The adhesion results indicated that cells adhered well on all the tested substrates. Nevertheless, phase contrast images and SEM analysis revealed differences in cell morphology depending on whether the cells were cultured on a 2D or 3D support. During culture on flat surfaces, MSCs formed a confluent monolayer, with spread and flat cells covering the available surface (Figure 2A,D). Differently from 2D culture, cells seeded on the 3D Nichoid penetrated into the internal structure of the niche and exhibited an elongated morphology and well distributed organization in all the three dimensions (Figure 2B,C,E,F). This spatial conformation allowed the cells to establish connections with both the surrounding cells and the niche’s 3D structure.

### 3.3. MSC Growth and Proliferation

**Figure 3:**
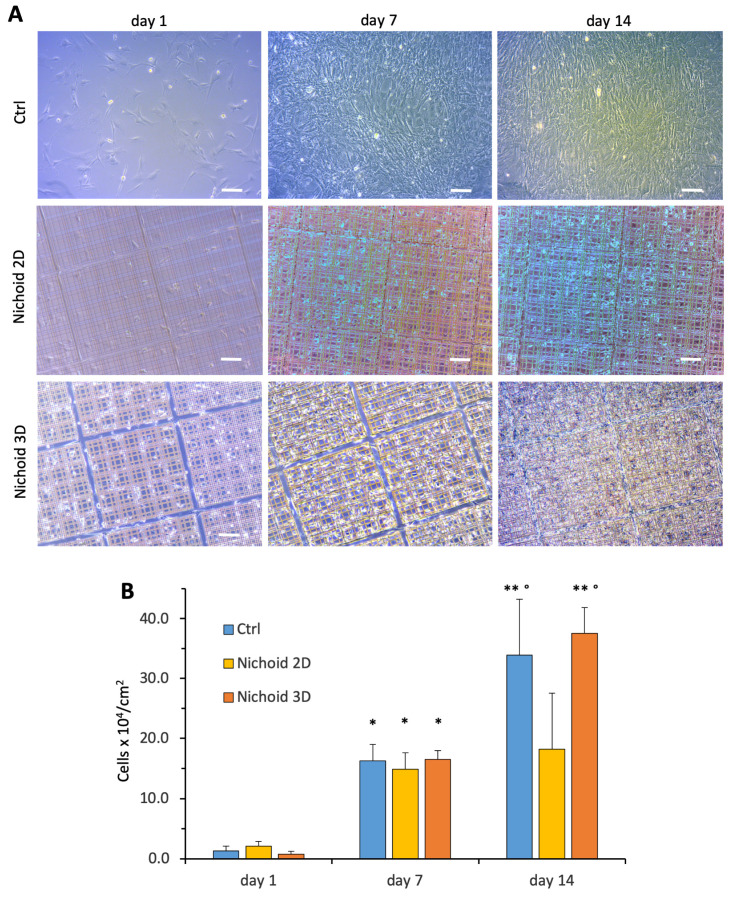
MSCs expansion on control and Nichoid structures. (**A**) Live images of rat MSCs cultured in control monolayer (upper panels), on the 2D Nichoid (middle panels) and on the 3D Nichoid (lower panels). Bars represent 100 µm. (**B**) Cell number × 10^4^ per cm^2^ after cell culture in flat control monolayer (Ctrl) or in the 2D Nichoid and 3D Nichoid substrates, (number of replicates *n* = 3–4 for each group). Data are presented as mean ± SD. * *p* < 0.01 vs. same group at Day 1, ** *p* < 0.01 vs. same group at Day 14 and ° *p* < 0.01 vs. 2D Nichoid (ANOVA and Bonferroni’s correction for multiple comparisons).

To evaluate growth and proliferation of MSCs during culture on flat control substrates and in the 2D and 3D Nichoid, we expanded cells for up to 2 weeks. Live images of cell culture revealed that the cells seeded on the flat substrate (Figure 3A, upper panels) reached confluence in about one week and continued to grow, with MSCs superimposed on the cell monolayer. MSCs cultured in the 2D Nichoid showed comparable proliferation to cells in control flat surface (see Figure 3A middle line) during the first week, but then they stopped proliferating. On the contrary, MSCs seeded in the 3D Nichoid showed cell proliferation comparable to flat substrate during the two weeks of culture (Figure 3A, lower panels), demonstrating that the 3D Nichoid is suitable for MSCs adhesion and growth.

Cell proliferation was evaluated by assessing the number of cell nuclei in flat controls and in the two Nichoid substrates at days 1, 7 and 14. From day 1 to day 7, cell proliferation was comparable among the three experimental conditions (see Figure 3B). We calculated, from cell density at day 1 and day 7, that cell doublings were comparable and on average equal to 3.6 ± 0.7, 2.8 ± 0.1 and 4.4 ± 0.2, respectively for cells on flat surface, on 2D and 3D Nichoids. During the following week of culture, MSCs on flat surface and on the 3D Nichoid continued to grow, even if at a lower rate, while cells on the 2D Nichoid showed almost no proliferation. Calculated cell doublings from day 7 to day 14 averaged 1.1 ± 0.07, 0.3 ± 0.24 and 1.2 ± 0.04 respectively for cells on flat surface, on the 2D Nichoid and on the 3D Nichoid (*p* < 0.05 2D Nichoid vs. flat controls and 3D Nichoid). We also verified by a live/dead staining (Live/Dead Viability/Citotoxicity Kit, Invitrogen detection technologies) that the cells were almost completely viable up to 14 days in the flat surface as well as on the 3D Nichoid (Supplementary material, Figure S3).

### 3.4. Focal Adhesion Contacts

**Figure 4:**
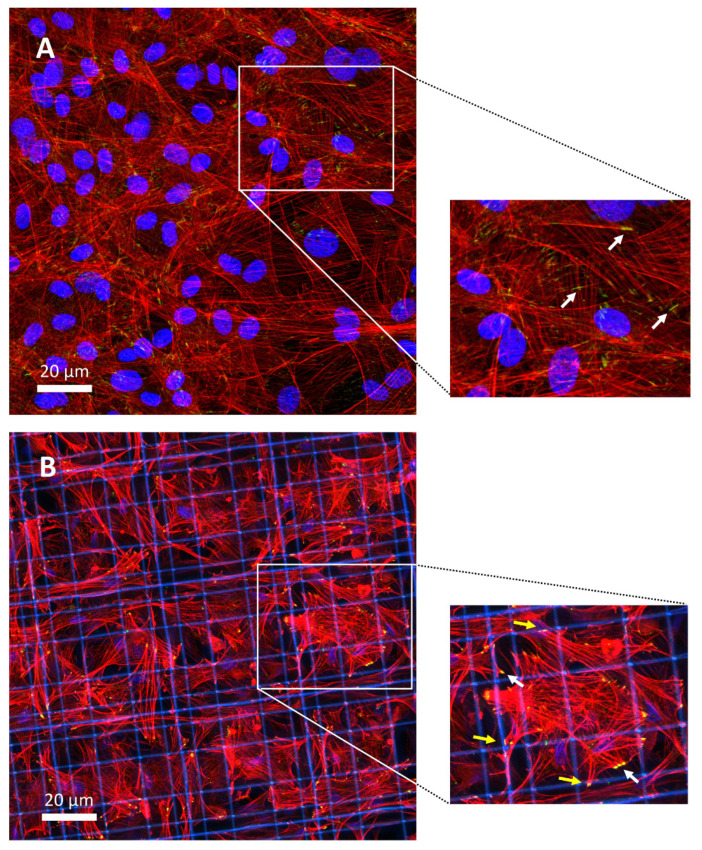
Immunofluorescence staining to F-actin and pFAK in MSCs in flat control monolayer (**A**) and in the 3D Nichoid (**B**). Focal adhesion contacts (yellow staining as merge of pFAK green staining and F-actin red staining) are highlighted by white arrows for adhesion of MSCs on flat surface (Inset A). In MSCs on 3D Nichoids, focal adhesions are present on cells adherent on flat substrate (Inset B—white arrows) and also on the Nichoid structure (Inset B—yellow arrows).

MSC adhesion is related to the expression of focal adhesions (FA) through which mechanical force influences regulatory signals. We then investigated the presence and location of FAs expression by MSCs on flat and 3D Nichoid substrates. As shown in Figure 4, cells grown on flat controls express pFAK complexes that are linked to F-actin fibers and mediate cell adhesion and spreading (see Figure 4A). Of interest, also MSCs cultured on 3D Nichoid show the presence of FAs, that are present in areas where cells are in contact with flat glass but also in areas of cell contact with the 3D Nichoid substrate (see Figure 4B). In these images, the F-actin fibers appear larger in size in MSCs grown in the 3D Nichoid as compared to control cells grown on flat surface, suggesting a different organization and dynamics of stress fiber assembly.

### 3.5. Cell Motility

**Figure 5:**
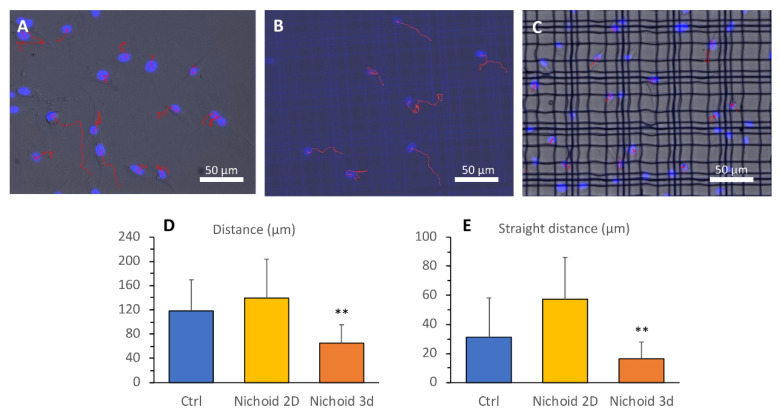
Cell motility. Immunofluorescence images and tracking of cell movements during 7 h acquisition in flat control monolayer (**A**), in the 2D Nichoid (**B**), and in the 3D Nichoid (**C**). (**D**) Quantification of the cell distance (or path, calculated as the sum of all the distances travelled) and (**E**) of the cell straight distance (or displacement, calculated as the net distance from the initial position to the final position) of cells cultured on flat control monolayer (Ctrl), 2D Nichoid and 3D Nichoid substrates (number of replicates *n* = 4 for each group, with total number of cells analyzed ranging from 38 to 60). Data are presented as mean ± SD, ** *p* < 0.001 vs. Ctrl and vs. 2D Nichoid.

We also assessed how the tested substrates affect MSCs motility by analyzing cell movements on the different culture settings. After 24 hours’ adhesion, cell movements were recorded by capturing fluorescence and phase contrast images every 5 min. Representative images of cell tracking during culture on flat control substrates or in the 2D and 3D Nichoid are displayed in Figure 5A–C, respectively, while movies on cell motility are shown in Supplementary Movie 1 and 2, in which one image was captured each 5 min. The analysis of cell movements revealed that flat control substrates allow cells to migrate and explore the surroundings, and cells on the 2D Nichoid showed comparable speed and straight distance. On the contrary, cells on the 3D Nichoid showed lower distance travelled, as well as lower straight distance (see Figure 5D,E). On this substrate, MSCs displayed a dynamic interaction with structural elements of the 3D Nichoid grid. Moreover, they preferentially remained within the structures without leaving their initial position. Since cells remain within the focus plane, their vertical displacement is likely limited (<10 µm).

### 3.6. Gene Expression Profile

**Figure 6:**
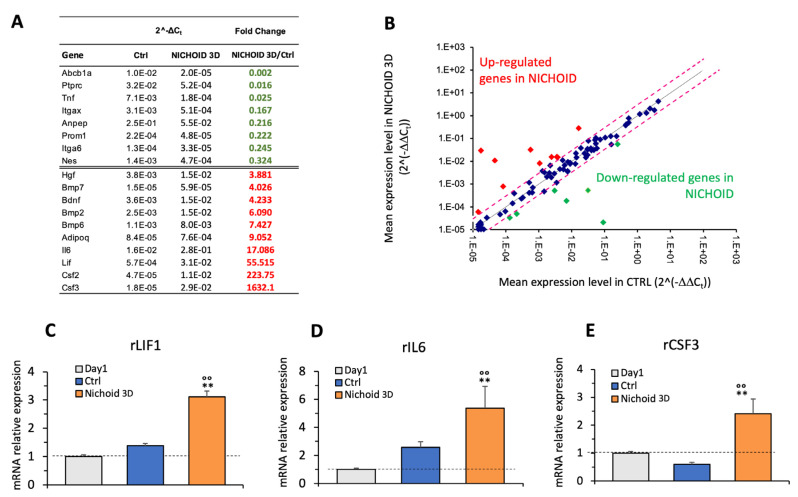
Gene expression analysis of MSCs cultured on the 3D Nichoid substrate. (**A**) Table of up- or down-regulated genes. (**B**) Scatter plot graph showing the expression level ((2^(-DDCt)) of each gene in the 3D Nichoid sample versus the flat control monolayer. The black line indicates the fold changes of 1, the pink lines indicate the fold changes of 2. Real-time PCR analysis for the expression of rLIF1 (**C**), rIL6 (**D**) and rCSF3 (**E**) in cells at day 1 (Day1) and in cell expanded for 2 weeks in the substrates (Ctrl and 3D Nichoid, *n* = 3 for each condition). Data are presented as mean ± SD, °° *p* < 0.01 vs. Day1, ** *p* < 0.01 vs. Ctrl (ANOVA and Bonferroni’s correction for multiple comparisons).

We next investigated whether culturing MSCs in the 3D Nichoid could induce changes in gene expression profiles. We examined, by a profiler PCR array, the expression of a panel of 89 key genes (see Gene table, Supplementary material). We used the RT2 profiler PCR array (PARN-082ZC-6 SABioscience, Qiagen, Frederick, MD, USA) because it makes it possible to profile genes related to rat MSC stemness, MSC-associated genes, and MSC differentiation markers involved in osteogenesis, adipogenesis, chondrogenesys, myogenesis and tenogenesis. We compared expression levels of these genes after 2 weeks of culture in two experimental conditions and we established that 18 out of 89 tested genes were significantly up- or down-regulated in 3D Nichoid-cultured MSCs, as compared to flat-cultured cells (see Figure 6A,B). Among the up-regulated genes, we selected three key genes related to MSC stemness and biological function to be validated by Real-Time PCR (see Figure 6A). We demonstrated that rLIF1, a pleiotropic cytokine that maintains the stem state of MSCs, was significantly higher in cells cultured in the 3D Nichoid, as compared to cells before seeding (Day1) and to cells expanded on flat control substrates (Figure 6C). Similarly, the expression of interleukin-6 (rIL-6), a cytokine that may contribute to the maintenance of MSCs in their undifferentiated state [31] was significantly higher in MSCs cultured in the 3D Nichoid substrate (Figure 6D). Moreover, we demonstrated a strong up-regulation of colony stimulating factor 3 (rCSF3) in the 3D Nichoid (Figure 6E) as compared to cells expanded on flat substrates.

### 3.7. Immunofluorescence and Western Blot Analyses

**Figure 7:**
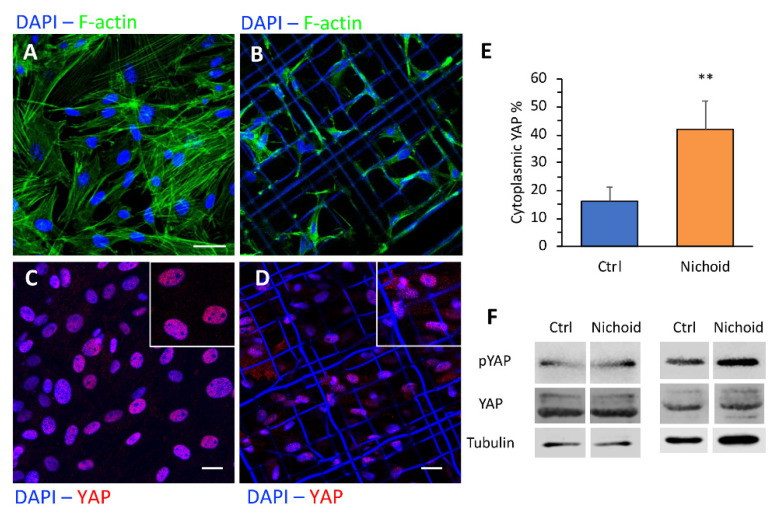
Cytoskeletal organization and YAP1 distribution. Immunofluorescence staining to F-actin (**A**,**B**) and YAP1 (**C**,**D**) of rat MSCs cultured in flat control monolayer (Ctrl) or in the 3D Nichoid for 7 days. Scale bars 20 µm. (**E**) Quantification of the cytoplasmic fraction of YAP positive area. (**F**) Immunoblotting of YAP1, p-YAP and tubulin expression in MSC cultured for 7 days in Ctrl an in the 3D Nichoid of two independent experiments. Data are presented as mean ± SD, ** *p* < 0.01 (*n* = 3, Student’s *t* test).

To gain insight into the mechanism by which the 3D substrate affected MSC gene expression, we explored organization of the actin cytoskeleton and localization of the mechano-transducer YAP [30]. Immunofluorescence staining for the actin cytoskeleton revealed that culturing MSCs in the 3D Nichoid resulted in remodeling of the actin organization. MSCs expanded on flat displayed large and flattened cytoplasm and nuclei (Figure 7A), with distinct F-actin stress fibers partly organized in parallel along the monolayer plane. Conversely, when cells were cultured in the 3D Nichoid substrate, they exhibited fewer long F-actin filaments, organized in three dimensions, and smaller spherical nuclei (Figure 7B). We then assessed whether the different mechanical signals perceived by cells cultured on the 2D and 3D substrate, by modifying the cytoskeleton organization, resulted in changes in localization of YAP, a key mediator of the mechanotransduction process. Immunofluorescence images showed that YAP was predominantly localized in the nucleus, independently from the substrate (Figure 7C,D). Nevertheless, quantification of YAP localization revealed that cytoplasmic YAP percentage was significantly increased in MSCs cultured in the 3D Nichoid as compared to flat-cultured cells (Figure 7E).

We investigated the effect of the different culture conditions on the expression of the YAP protein and that of phosphorylated YAP. Quantification of the Western blot bands (reported in Figure 7F) allow to estimate that the expression of YAP did not change significantly between cells cultured on flat controls (Ctrl) as compared to cells cultured in 3D Nichoids (4% decrease, *n* = 2). On the contrary, expression of pYAP increased in the 3D Nichoid substrate, on average of 27% (*n* = 2).

### 3.8. MSC Ultrastructural Analysis

**Figure 8:**
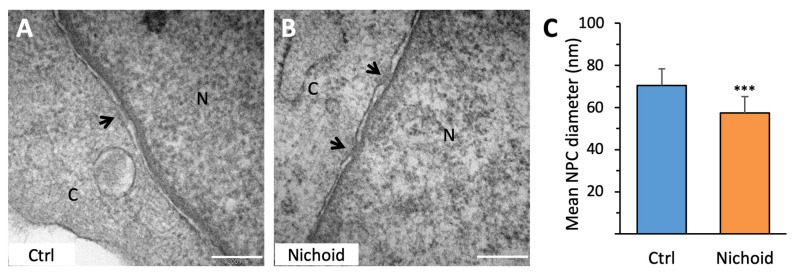
Ultrastructural analysis of the nuclear envelope. Transmission electron microscopy (TEM) images of MSCs cultured on flat control monolayer (**A**) or in the 3D Nichoid (**B**) for 7 days. N, nucleoplasm; C, cytoplasm. Black arrows indicate nuclear pores. (**C**) Quantification of mean nuclear pore diameter in MSCs expanded on control monolayer or in the 3D Nichoid. Bar represents 200 nm. Data are presented as mean ± SD, *** *p* < 0.001 (Student’s *t* test).

After observing the above mentioned alterations in cell shape and organization, we extended our analysis up to the ultrastructural level, in order to establish whether mechanical deformation of the cell was transmitted to the nucleus. TEM analysis allowed the observation of the nuclear envelope of cells cultured on 2D or 3D culture settings and identification of nuclear pores. TEM images showed the presence of nuclear pores in both experimental conditions, identified by a dense area flanked by two adjacent segments of nuclear envelope (Figure 8A,B). The quantification of the mean diameter of nuclear pores in the different culture substrates revealed that the flattened morphology of cells cultured on flat control substrates induced stretch of the nuclear envelope and the increase of nuclear pore diameter (70.5 ± 7.9 nm). Conversely, MSCs cultured in the 3D Nichoid exhibited more spherical nuclei and had smaller nuclear pores, with a mean diameter of 57.3 ± 9.7 nm (Figure 8C).

## 4. Discussion

MSCs are the preferred source for stem cell transplantation to induce organ regeneration or transplant tolerance. The procedure of cell harvesting and the possibility of transplanting non-autologous cells makes this cell therapy feasible in the clinical setting. The clinical results of MSC treatment are still not completely assessed. There is evidence that MSC may induce some organ function recovery [32,33] and induce graft immune tolerance in organ transplantation [34,35,36]. The mechanisms responsible for these effects, however, are not well understood so far. It is then important to establish which are the factors that induce MSC phenotype change in vitro, a stage necessary for in vivo transplantation of these cells in patients, not only to obtain the best performing stem cell for implantation, but also to understand the processes that take place during MSC cell homing in different tissues and the following effects. We focused our investigation on the effect of 3D culture of MSC in a peculiar micro-structure, as compared to culture conditions based on a flat 2D surface, which is the conventional cell substrate used for MSC expansion before cell implantation.

There is evidence that cell deformation and mechanical forces perceived by cells in 2D conditions result in nuclear envelope stretch that affects nuclear transport [37,38]. This culture condition is also known to induce MSC differentiation and, to a certain degree, loss of cell stemness [12]. We have previously shown that stemness of embryonic stem cells (ESCs) and MSCs can be better maintained by their culture on the 3D micro-structure used here [28,39]. Here we have investigated more in depth the effects of this 3D culture system on cell biological functions. We obtained new findings comparing cell cultures grown on flat substrates, on a one-level Nichoid and on the 3D Nichoid micro-structure. This allowed us to isolate the cue related to tridimensional architecture of the Nichoid and to demonstrate more convincingly the effect of this spatial disposition on important aspects of MSC response. In the present investigation, we also estimated aspects of MSC response quantitatively on millions cells, all cultured in the 3D Nichoids, instead of only qualitatively on a few thousands of cells cultured in sparse Nichoids, covering only 10% of the total culture surface, as done previously [26,27]. Using these methods, we could demonstrate mainly an effect of the Nichoid on guiding MSC homing and reducing lineage commitment, as compared to flat culture. Later, previous data demonstrated an increased multipotency induced in MSC by the Nichoid [28,40] but this effect was inevitably diluted by the cells that did not experience the 3D environment, i.e., those deposited on the glass surface surrounding the Nichoids. As such, flat-cultured cells could not be separated from those localized in the Nichoids, so that the observed differences were significant but not substantial. In the present investigation, by using greater culture surface and 3D Nichoid coverage (>90% of the surface area), we were able to culture almost all the cells inside the 3D Nichoid and to harvest millions of cells and thus to characterize the substantial effect of the Nichoid on up regulation of several genes related to MSC stemness and function. This demonstration required a considerable technological effort [25]. In this regard, by focusing the two-photon laser simultaneously on six different points on the same sample, we recently managed to reduce the Nichoid fabrication time to 2.5 h/sample [41]. After a huge technological effort in the up-scaling of the laser fabrication technique, lasted ten years, we are now able to obtain enough expanded cells from the 3D Nichoid substrate to further increase the robustness and the quality of our future observations. This achievement may also open an avenue towards the production of clinically significant cell doses, in view of the translation of MSC-based cell therapies.

We documented that MSCs grow in the 3D Nichoid system similarly to cells grown on flat surfaces. The large number of MSCs estimated after 14 days in culture in the 3D Nichoid can benefit from the large volume available for cell attachment with a 30 µm in height. Interestingly, MSCs on the 2D Nichoid (see Figure 3A) showed a lower proliferation rate during the second week in culture, suggesting that the presence of the corrugated surface, and/or the material of the grid itself, interfered with cell proliferation. These data confirm that the 3D environment of the Nichoid favorably affected cell division, despite the general thinking is that cells on a flat and rigid substrate undergo cell division more efficiently. Of note, the lower proliferation rate of MSCs day7–day14 period on the 2D Nichoid may be due to the confluence of cells and cell contact that may inhibit further cell division. These data are in line with our previous findings on human MSCs proliferation in the Nichoid system [28]. The 3D artificial niche may allow cells to adhere and proliferate, and the Nichoid micro-grid was also found to significantly confine cell migration. As expected, we documented a different effect of 2D and 3D substrates on cell movement. While MSCs cultured on a flat surface or on the 2D Nichoid migrate over long displacements, cells in the 3D Nichoid display shorter overall displacements and shorter paths. It is unlikely that the shorter distance measured is due to possible cell movements in the vertical direction, as cells were always in focus during time-lapse microscopy. In line with previous evidence, this could be due to a more difficult spatial reorganization of adhesion complexes and of the actin cytoskeleton that may influence cell migration [42].

We then investigated whether the different mechanical forces that develop along the cytoskeleton structure may induce changes in transmitting forces from the focal adhesion sites to the nuclear membrane. Our data show that FAs are present where cells are in contact with the 3D Nichoid structure, as revealed by expression of F-actin fibers and pFAK in these sites. The focal adhesion sites of MSCs in the 3D space may mediate cell signaling induced by mechanical forces. In addition, our immunofluorescence images of F-actin fibers show larger stress fibers in MSCs grown on 3D Nichoid, as compared to cells on flat controls, suggesting difference among the two experimental conditions in formation of F-actin bundles. Recently, it has been reported that F-actin bundling enhances glycolysis, suggesting that this as an important pathway for mechanotransduction [37]. We also hypothesized that, for the 3D distribution of mechanical forces, nuclear pore dimensions may change in cells grown in flat controls, as compared to 3D Nichoid substrates. Our results, based on high-resolution TEM, clearly show that this is actually the case and that nuclear pore mean diameter was 20% higher in cells spread on flat surface, as compared to cells uniformly distributed in the 3D Nichoid space. It has been recently demonstrated that intracellular forces generated by the cytoskeleton in cell spreading on rigid flat surfaces induce nuclear flattening, nuclear pore stretching and increased molecular transport from the cytoplasm to the nucleus [38]. Thus, in MSCs cultured in the 3D Nichoid substrate, smaller nuclear pores could reduce the transport of molecules from the cytoplasm to the nucleus. In particular, this effect may be relevant for the transfer to the nucleus of transcriptional factors.

Indeed, we documented that the 3D culture condition affected cellular distribution of the transcriptional co-activator YAP, with higher expression in the cytoplasm, as compared to flat-cultured cells, which showed major nuclear location of YAP. Western blot quantification allowed to document an increased expression of pYAP in cells cultured in the 3D Nichoid. The effect of mechanotransduction on YAP nuclear translocation in MSCs cultured on substrates with different stiffness has already been demonstrated [30,43]. Our results show, for the first time, that in addition to stiffness, a substrate that induces 3D cell organization can also be responsible for changes in YAP nuclear translocation. In detail, the evidence that nuclear pores are smaller in diameter in MSCs cultured in 3D suggests that steric hindrance may be the mechanism responsible for lower YAP transport to the nucleus, with higher content of pYAP, likely that confined into the cytoplasm [44]. In conventional cell culture substrates, due to cell spreading and the related action of cytoskeleton (connecting focal adhesions to the nuclear envelope) nuclear pores are enlarged and they allow the transport of the mechano-transducers YAP/TAZ and other transcription factors into the cell nucleus more easily, triggering activation of genetic programs that ultimately result in biological response. Since these transcription factors affect gene expression, it is important to understand how cell anchorage to the external structures may induce cytoskeleton organization, that in turn directly affects the function of factors responsible for gene expression regulation. In addition, whether the change in size in nuclear pores may also change nuclear translocation of other transcription factors, and how, is worth to further investigate.

Our data demonstrate that cell culture in the 3D Nichoid system strongly affects the expression of 18 over the 89 genes tested. Among these genes, we verified by real-time PCR that expression of three key genes involved in MSCs stemness and function is significantly influenced by the 3D cell shape. Actually, the 3D substrate induced the upregulation of LIF1, a pleiotropic cytokine involved in stemness maintenance of MSCs and of other stem cells [45]. Moreover, the expression levels of IL6, a cytokine related to the undifferentiated state of MSCs whose expression decreases during cell differentiation [31], are significantly higher in cells cultured on the 3D artificial niche. Finally, we documented a strong up-regulation of gene expression of the hematopoietic factor CSF3. This factor is also recognized as a neuronal ligand that is able to counteract cell death and exhibits neuroprotective mechanisms [46,47]. Our findings of an increased expression of cytokines in 3D Nichoid-cultured MSCs are in line with other investigations demonstrating an enhanced paracrine and pro-angiogenic effect of 3D cultured MSCs [48,49,50]. Our data also indicate how the 3D spatial disposition of the cells, once implanted in different tissues, may be responsible for MSCs favorable effects during tissue repair processes. On the contrary, when cells grow on flat surfaces they behave differently, and one additional reason could be the inhibition of cell biological functions induced by cell contact.

It is of interest that among the genes that are differentially expressed by the two culture conditions we studied, some of them are regulated by YAP. In particular, Tnf, which we estimated to be downregulated in MSCs culture in the 3D Nichoid, are known to be regulated by YAP [51]. As far as genes upregulated by the 3D Nichoid, as compared to the flat surface, Lif, IL6, and BMPs are also known to be regulated by YAP [52,53,54]. These observations further corroborate the evidence that 3D geometric disposition of the cells, and their interaction with the substrate effectively induce changes in gene expression mediated by the different action of the transcription factor YAP. Furthermore, compared to flat controls, in 3D Nichoid-cultured cells we measured not only possessed an increased retention of cytoplasmic YAP, known to affect the cell doubling rate [30,42], but also up-regulation of BMP2 and BMP7 expression (Figure 5A) known to stimulate proliferation in MSC [55]. The 3D Nichoid is likely sensed by cells as a stiff environment in all the 3D space surrounding the cells, it is thus more effective than conventional flat substrates in up-regulating the expression of stiffness-mediated genes such as BMP7 [12]. The YAP and BMP7 signals likely compensate each other in 3D Nichoids versus flat controls, resulting in comparable proliferation rates, despite the general thinking that cells on a flat and rigid substrate undergo cell division more efficiently.

## 5. Conclusions

Our results show that MSCs grown in the 3D Nichoid microenvironment are influenced by the spatial distribution of cell volume and focal adhesion sites, reducing cell motility, and affecting gene expression, nuclear pore dimensions as well as the cytoplasmic YAP. These mechanobiology effects may be useful for preserving MSCs stemness and function during in vitro culture to potentially obtain more favorable effects in cell therapy. At the same time, our findings indicate a potential mechanism for the regulation of MSCs function in vivo, as implanted cells are disposed in 3D in the hosting tissue, releasing trophic and protective factors that act on surrounding cells and are necessary for generating effective tissue repair.

## Supplementary Materials

The following are available online at https://www.mdpi.com/2073-4409/9/8/1873/s1, Supplementary material (.pdf 4.8 Mb) and Supplementary movie 1 (.mov 4261 kb) and 2 (.mov 4261 kb).
